# Draft Genome Sequence of the *Microbispora* sp. Strain ATCC-PTA-5024, Producing the Lantibiotic NAI-107

**DOI:** 10.1128/genomeA.01198-13

**Published:** 2014-01-23

**Authors:** Margherita Sosio, Giuseppe Gallo, Roberta Pozzi, Stefania Serina, Paolo Monciardini, Agnieska Bera, Evi Stegmann, Tilmann Weber

**Affiliations:** a Naicons S.r.l., Milan, Italy; b Ktedogen S.r.l., Milan, Italy; c Department of Biological, Chemical and Pharmaceutical Sciences and Technologies, University of Palermo, Palermo, Italy; d Interfaculty Institute of Microbiology and Infection Medicine Tübingen, Eberhard Karls University, Tübingen, Germany

## Abstract

We report the draft genome sequence of *Microbispora* sp. strain ATCC-PTA-5024, a soil isolate that produces NAI-107, a new lantibiotic with the potential to treat life-threatening infections caused by multidrug-resistant Gram-positive pathogens. The draft genome of strain *Microbispora* sp. ATCC-PTA-5024 consists of 8,543,819 bp, with a 71.2% G+C content and 7,860 protein-coding genes.

## GENOME ANNOUNCEMENT

*Microbispora* sp. strain ATCC-PTA-5024 is a Gram-positive, aerobic, filamentous actinobacterium. It is the producer of NAI-107, a new lantibiotic that is active against all Gram-positive pathogens, including multidrug-resistant isolates, with good potency and efficacy in sophisticated experimental models of infection, such as rat endocarditis caused by methicillin-resistant *Staphylococcus aureus* (1).

This announcement reports the derivation of data to enable the generation of a scaffold genome sequence for this important industrial strain.

Genome sequencing of *Microbispora* sp. ATCC-PTA-5024 was carried out using a combination of 454 (2) and Illumina (3) technologies. In a first run, a total of 173,473 reads were generated on a Roche/454 GS FLX system, yielding 56.6 Mb of sequence. Assembly by Newbler was possible for 89% of the reads, yielding 4,227 >500-bp contigs and 190 scaffolds, with the largest scaffold being 306,421 bp. The total length of the scaffolds was 8,091,813 bases. Subsequently, 423,880 reads were generated in a second 454 run from a library of random and 3.5-kb paired-end fragments, giving a total of 124.2 Mb of sequence (ca. 16× coverage). Assembly by Newbler was possible for 91% of the reads, yielding 847 >500-bp contigs and 8 scaffolds. The Illumina GAII shotgun library generated 3.25 million reads totaling 813.6 Mb. A hybrid assembly combining the 454 and Illumina data with Newbler 2.6 resulted in a decreased number of 371 contigs of >500 bp and a genome size of 8,543,819 bases.

Many scaffolds show extensive synteny with the complete genome of *Streptosporangium roseum*. This was used to link seven out of eight scaffolds and create *in silico* a superscaffold of 8,407,907 bp in length. The scaffolds of the *Microbispora* sp. strain ATCC-PTA-5024 genome were analyzed using the NCBI PGAP version 2.1 (http://www.ncbi.nlm.nih.gov/genomes/static/Pipeline.html).

The draft genome of *Microbispora* sp. ATCC-PTA-5024 is estimated to have a total of 7,860 protein-coding genes, along with 58 tRNAs. The genomic data include about 200 contigs, spanning a total of 0.1 Mb, which were not assembled into scaffolds. These unassembled contigs include mostly repeated sequences, such as *rrn* operons and insertion sequences, which are likely present in multiple copies in the genome. Some of these unassembled contigs can be used to fill in gaps in regions of interest, using a reference sequence as a template. For example, the *Microbispora* sp. strain ATCC-PTA-5024 genome, like that of the related genus *Nonomuraea* (4), contains two *rpoB* alleles, with an identical central 1,774-nucleotide (nt) portion that could not be assembled by Newbler. Also, the regions flanking the *mlb* cluster, which are required for NAI-107 biosynthesis (S. Donadio, M. Sosio, S. Serina, and D. Mercorillo, 12 February 2009, PCT patent application WO 2009/019524), are enriched in glycosidases and were joined to the *mlb* cluster using the related cluster from *Microbispora corallina* (5).

To identify secondary metabolite gene clusters, the analysis pipeline antiSMASH was run on the genome (6, 7), giving an overview on the secondary metabolite potential of this strain. A total of 20 potential clusters for secondary metabolites were identified in the *Microbispora* genome, in addition to the *mlb* cluster.

### Nucleotide sequence accession number.

This whole-genome shotgun sequence has been deposited at GenBank under the accession no. AWEV00000000.
